# Deciphering a subgroup of breast carcinomas with putative progression of grade during carcinogenesis revealed by comparative genomic hybridisation (CGH) and immunohistochemistry

**DOI:** 10.1038/sj.bjc.6601658

**Published:** 2004-03-02

**Authors:** E Korsching, J Packeisen, M W Helms, C Kersting, R Voss, P J van Diest, B Brandt, E van der Wall, W Boecker, H Bürger

**Affiliations:** 1 Institute of Pathology, University of Münster, Domagkstr. 17, 48149 Münster, Germany; 2 Institute of Pathology, Klinikum Osnabrück, Domagkstr. 17, 48149 Münster, Germany; 3 Laboratory Medicine, Institute of Clinical Chemistry, University of Münster, Domagkstr. 17, 48149 Münster, Germany; 4 Institute of Atherosclerosis Research, University of Münster, Domagkstr. 17, 48149 Münster, Germany; 5 Institute of Pathology, Utrecht University Medical Center, The Netherlands; 6 Department of Medical Oncology, Utrecht University Medical Center, The Netherlands

**Keywords:** breast cancer, CGH, progression, grade

## Abstract

Distinct parallel cytogenetic pathways in breast carcinogenesis could be identified in recent years. Nevertheless, it remained unclear as to which tumours may have progressed in grade or which patterns of cytogenetic alteration may define the switch from an *in situ* towards an invasive lesion. In order to gain more detailed insights into cytogenetic mechanisms of the pathogenesis of breast cancer, the chromosomal imbalances of 206 invasive breast cancer cases were characterised by means of comparative genomic hybridisation (CGH). CGH data were subjected to hierarchical cluster analysis and the results were further compared with immunohistochemical findings on tissue arrays from the same breast cancer cases. The combined analysis of immunohistochemical and cytogenetic data provided evidence that carcinomas with gains of 7p, and to a lesser extent losses of 9q and gains of 5p, are a distinct subgroup within the spectrum of ductal invasive grade 3 breast carcinomas. These aberrations were associated with a high degree of cytogenetic instability (16.6 alterations per case on average), 16q-losses in over 70% of these cases, strong oestrogen receptor expression and absence of strong expression of p53, c-*erb*B2 and Ck 5. These characteristics provide strong support for the hypothesis that these tumours may develop through stages of well- and perhaps intermediately differentiated breast cancers. Our results therefore underline the existence of several parallel and also stepwise progression pathways towards breast cancer.

Technology for the molecular investigation of breast carcinogenesis has evolved tremendously in the last decade with the introduction of whole-genome assays, focusing on RNA (gene-expression analysis) (Alizadeh *et al*, 2001) and DNA levels within the cell (comparative genomic hybridisation; CGH) (Kallioniemi *et al*, 1992). In recent years, the use of CGH led to the proposal of an integrated morphological/cytogenetic progression model for breast cancer (Vos *et al*, 2000; Buerger *et al*, 2001) with at least two different, parallel cytogenetic pathways: the well-differentiated and the poorly differentiated pathways. The loss of 16q thereby seems to represent the cytogenetic hallmark of the well-differentiated progression pathway, even though a subset of poorly differentiated tumours also revealed 16q-losses. For these tumours, it remained unclear if they are the end stage of a de-differentiation process via well-differentiated cancers, or if in these cases 16q-losses are merely secondary events. Nevertheless, it also became clear that the majority of breast cancers and its ultimate precursor lesions are characterised by the presence of a distinct genetic alteration (Buerger *et al*, 1999a). Our understanding of carcinogenesis in general (Hanahan and Weinberg, 2000) and breast carcinogenesis specifically is challenged by the postulation of differential complex cytogenetic aberration (Buerger *et al*, 1999b) and RNA expression patterns (Perou *et al*, 2000). These postulates may enable a better explanation of genotype–phenotype correlations, and may lead to the establishment of new prognostic markers (van't Heer *et al*, 2002). The obvious increase of knowledge using these techniques is mirrored by the huge amount of data requiring complicated models for biomathematical analysis.

In this study, we would like to show that the re-evaluation of CGH-data by conventional and biomathematical analysis of 206 cases of invasive breast cancer cases provides further insights into cytogenetic events during breast carcinogenesis. These new aspects might close at least some gaps in our current understanding of breast cancer.

## MATERIALS AND METHODS

### Material

A total of 206 invasive breast cancer cases were staged according to the TNM-system. The tumour series represented all subtypes of invasive breast cancer, and the tumours were graded according to established protocols (Ellis and Elston, 1998) as G1 (*n*=32), G2 (*n*=97) or G3 (*n*=77). The validation of tumour grade has been achieved using consensus panels as previously described (Buerger *et al*, 2001). In addition, all ductal invasive G3 carcinomas have been subjected to morphometric analysis and the tumour grades revealed significant differences concerning tubule formation, nuclear size, and mitotic rate (Buerger *et al*, 2001).

### Methods

#### CGH-analysis

The method of CGH-analysis, the corresponding control experiments and the criteria for the evaluation of genetic alterations were performed as previously described (Kallioniemi *et al*, 1994; Buerger *et al*, 1999b). CGH was performed on fresh frozen tissue samples.

#### Biomathematical analysis

The CGH ratio profiles and IHC raw data were tabulated in a range from 1 to 2 and from 1 to 3, respectively. Missing IHC data (9% of all IHC data) were replaced by the median of that specific score. This procedure approximated the real values in a reasonable manner, and did not bias the evaluation (data not shown here). In the analysis of CGH data combined with IHC data, the scales of both categories were adapted to achieve equally weighted variables. The different lengths of the CGH and IHC data in the feature vector were not changed. To analyse the independent behaviour of the CGH and IHC feature vectors, both categories were also analysed separately. Hierarchical cluster analysis based on a Euclidean distance measure was applied (Alaiya *et al*, 2002; Harris *et al*, 2002) with the underlying and tested rationale that our case's set did not include extreme outliers. We used here ‘Complete Linkage’ as hierarchical cluster method, but we have tested the behaviour of similar methods (above all ‘Ward’). The comparative analysis of these further algorithms showed that the basic message in the result sets was the same. Therefore, we decided to choose the common method ‘Complete Linkage’ because of the comparability to other data. Our evaluations were performed with the statistical platform SPlus6.1-r2 using the functions ‘hclust’ and ‘agnes’ – based on algorithms as previously described (Kaufman *et al*, 1999; Struyf *et al*, 2002). The results are visualised in a dendrogram showing in a graphical way the similarity of the cancer cases. Coloured labels on a branch show the major characteristics of that case.

#### Flow cytometry

DNA ploidy analysis was performed on single-cell suspensions prepared from 50-*μ*m-thick paraffin samples with a PAS II (Partec Instruments, Arlesheim, Switzerland) mercury lamp-based flow cytometer. DNA histograms were analysed using the MultiCycle (Phoenix Flow Systems, San Diego, CA, USA) cell cycle analysis software according to the previously established protocols (Bergers *et al*, 1996, 1997).

#### Tissue microarrays and immunohistochemistry

A tissue microarray was constructed according to the standard procedures (Kononen *et al*, 1998), containing 153 invasive breast cancer cases that were representative of the whole tumour group and were fully characterised by CGH.

Immunohistochemical staining procedures for ER, PR, bcl-2, p21, Ck 5/6, Cyclin D1, Ki-67, p53, Cyclin A, p27 and c-*erb*B-2 have been performed as previously described. The pretreatment conditions, the source and the dilution of the commercially available primary antibodies, as well as the guidelines for a semiquantitative evaluation have been published elsewhere (Korsching *et al*, 2002).

In brief, expression was binary graded for ER and PR, bcl-2, p21, Cyclin D1, and Ck 5/6, Ck 8/18 and SMA. Expression was graded from 0 to 3 for Ki-67, p53, Cyclin A and p27 according to the percentage of positive cells. c-*erb*B-2 was classified according to the Dako-Score (Korsching *et al*, 2002).

## RESULTS

A detailed description of cytogenetic alteration patterns of the invasive breast cancer cases subjected to biomathematical analysis has been presented before (Buerger and Boecker, 2002). Further detailed characterisation is presented for ductal invasive grade 3 carcinomas only.

### DNA flow cytometric and cytogenetic characteristics of ductal invasive grade 3 breast cancer cases with 16q-losses

A total of 15.5 alterations per case were seen in ductal invasive grade 3 breast cancers with 16q-losses. Three out of 12 tumours (25%) were DNA diploid, one tumour was tetraploid; 66% were aneuploid. ER and PR were expressed in 70 and 54% of the cases, respectively. The most frequent chromosomal alterations were 1q-gains (84%), 3q-gains (50%), 5p-gains (33%), 7p-gains (41%), 8p-losses (64%), 8q-gains (84%), 9q-losses (58%), 11q-losses (50%), 13q-losses (58%), 15q-losses (33%), 17q-gains (33%) and 20q-gains (50%).

### Biomathematical modelling of cytogenetic alteration patterns in breast cancer

**Figure 1:**
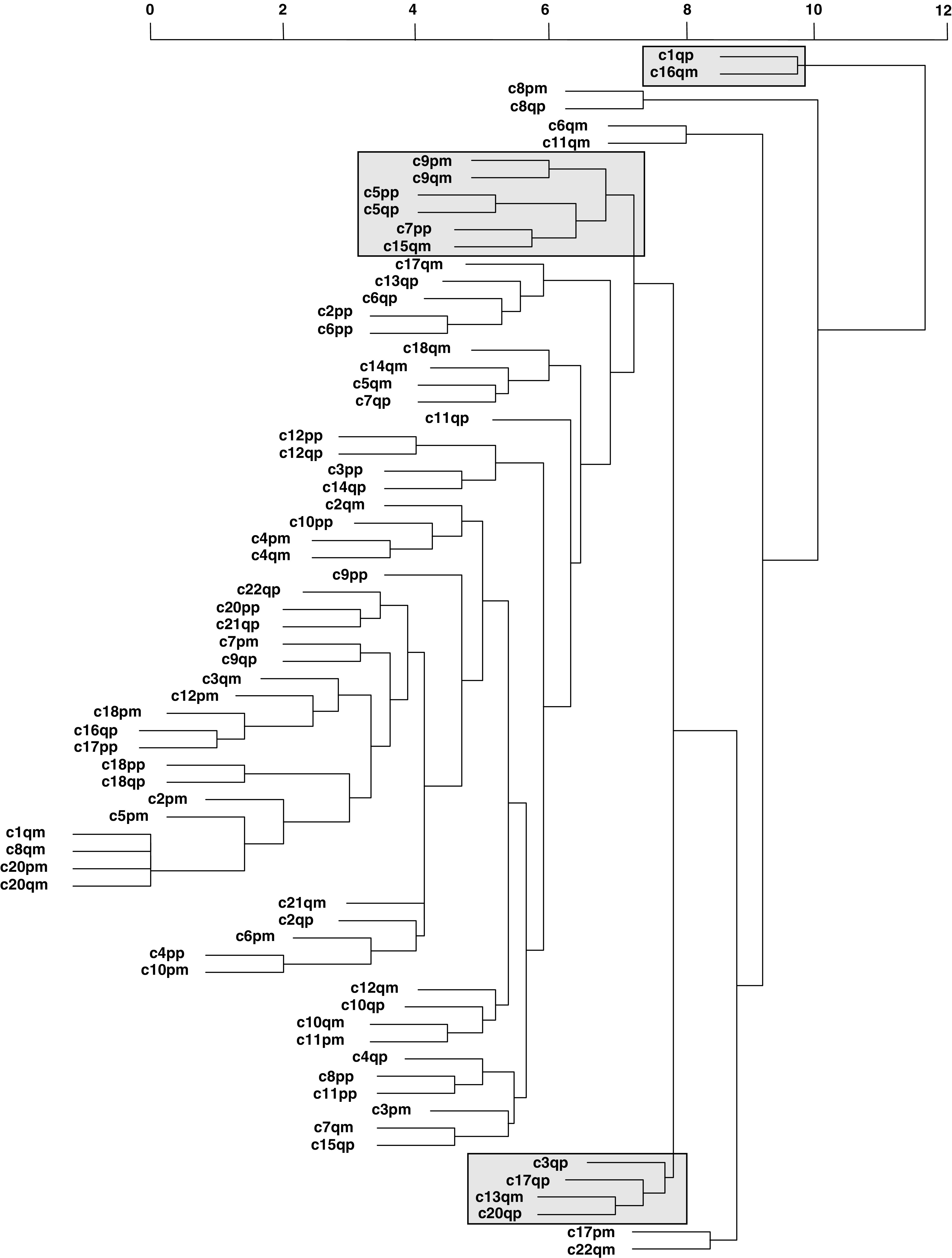
Dendrogram of cytogenetic alteration patterns revealed from CGH results from 206 invasive breast cancer cases. p and m indicate gains and losses of the short (p) and long (q) arm of the respective chromosome. Three clusters of interest are indicated by a frame. Whereas for example the 1q-gain/16q-loss cluster is indicative of highly differentiated tumours (Tsuda *et al*, 1997), two other clusters are indicative of high-grade carcinomas.

Biomathematical modelling revealed the presence of multiple clusters in invasive breast cancer as indicated in Figure 1

. Distinct clusters included, for example, 1q-gains/16q-lossses, 9q-losses/5p-gains/7p-gains/15q-losses or 3q-gains/17q-gains, 20q-gains and 13q-losses, respectively, besides a multitude of other clusters (Figure 1).

### Immunohistochemical characterisation of all ductal Invasive grade 3 carcinomas/with 16q-losses/with 7p-gains/with 5p-gains/with 9q-losses

An overview of the absolute numbers of the respective subgroups and the associated immunohistochemical findings in these subgroups is given in Table 1

**Table 1:** Overview of immunohistochemical staining patterns and incidence of 16q-losses in subgroups of ductal invasive grade 3 breast cancers

	**Grade3 all**	**Grade3 with 16q-losses**	**Grade3 with 7p-gains**	**Grade3 with 5p-gains**	**Grade3 with 9q-losses**
Average number of cytogenetic alterations per case	11.7	15.5	16.6	16.8	13.0
					
ER all (+) cases	58	70	84	75	66
PR all (+) cases	63	54	70	30	58
Mib-1 (++)–(+++)	90	90	100	100	90
c-*erb*B2 (−)–(++)	74	80	100	80	100
p53 (−)–(+)	85	100	100	80	81
Cyclin D1 all (+) cases	25	33	40	22	44
bcl-2 all (+) cases	24	60	60	44	50
p21 all (+) cases	41	50	60	45	40
p27 (−)–(++)	87	50	40	88	80
Cyclin A (++)–(+++)	75	80	100	90	72
Ck 5 all (+) cases	23	20	20	44	20
16q-losses	27	100	71	50	58

The frequencies are indicated in %. The average number of cytogenetic alterations per case is given in absolute numbers.

. Interestingly, for most of the markers a clear difference of the immuno-profile could be observed for the five different groups. Despite high proliferation indices (Mib-1 and Cyclin A) and the high degree of cytogenetic instability in ductal invasive grade 3 carcinomas with 7p-gains, the frequency of 16q-losses and the percentage of ER/PR+tumours were significantly higher compared to other subgroups. Similar data were obtained by the re-evaluation of ductal invasive grade 3 carcinomas with 5p-gains or 9q-losses, respectively, even though the percentage, especially of ER and PR+tumours, was lower (Table 1).

### DNA flow cytometric and cytogenetic characteristics of ductal invasive grade 3 carcinomas with 7p-gains

Since 7p-gains with 16q-losses were among the most frequent alterations, these ductal invasive grade 3 carcinomas were further characterised under this new perspective.

All these tumours were nondiploid, one was DNA tetraploid and the remaining were DNA aneuploid. On average, 16.6 CGH alterations per case were detected.

16q-losses, 9q-losses, 8q-gains and 1q-gains were present in more than 70% of these tumours. 5p-gains were detected with a frequency of 52%. In all, 84% of these tumours were oestrogen receptor positive and 70% were progesterone receptor positive. None of the tumours revealed a strong c-*erb*B2 or p53-accumulation. In all, 20% displayed a Ck 5/6 positive phenotype.

### Biomathematical clustering of immunohistochemical expression patterns in invasive breast cancer

**Figure 2:**
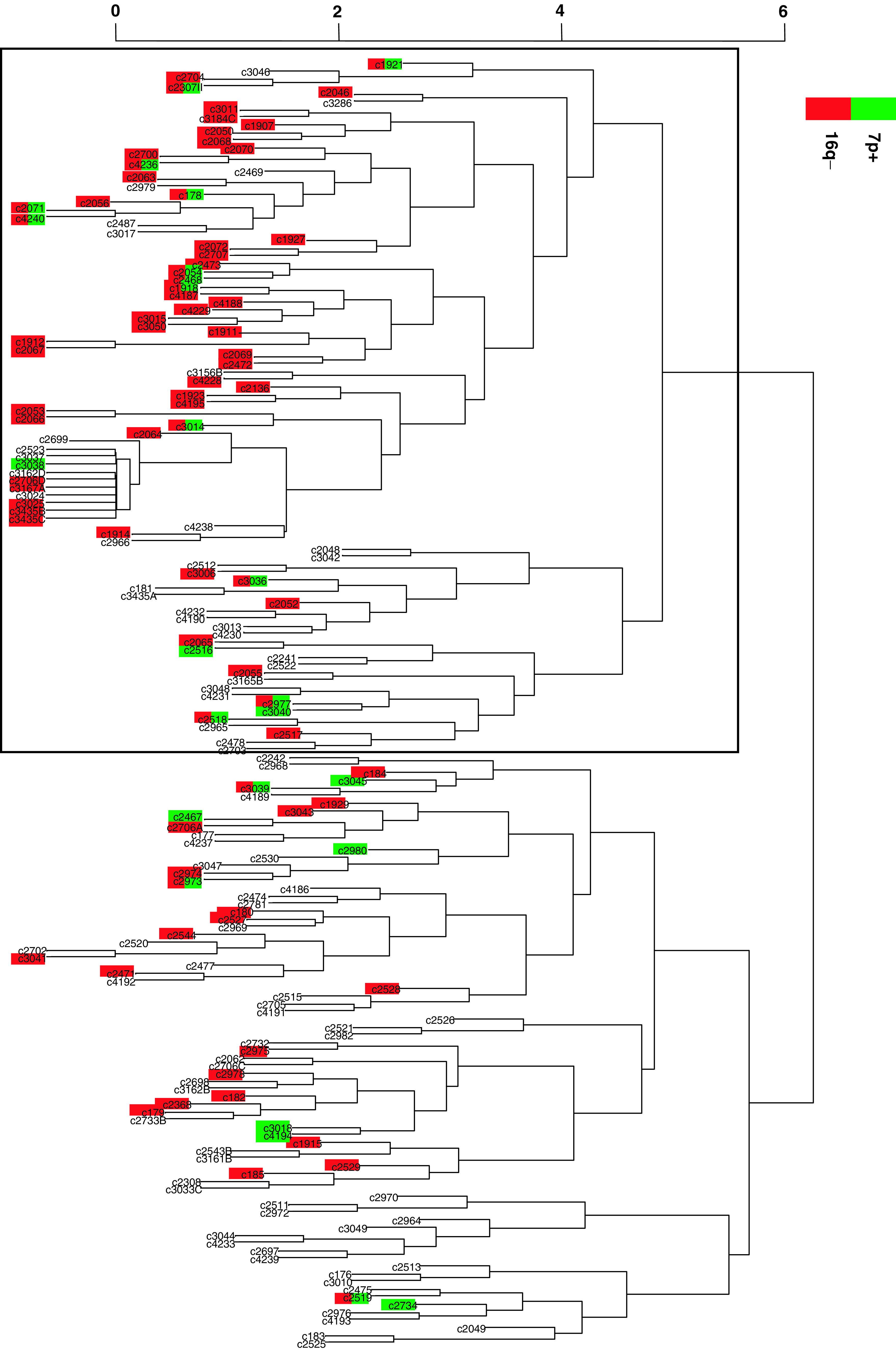
Dendrogram of 153 invasive breast cancer cases, clustered by their protein expression patterns. Two major clusters were formed. One cluster arm (indicated by a frame) is characterised by a significantly higher number of 16q-losses and 7p-gains. 16q-losses and 7p-gains are indicated in red and green, respectively.

Two major cluster arms could be identified (Figure 2

). One of these major cluster arms (indicated by a frame in Figure 1) revealed a lower average number of genetic alterations per case (7.68±6.08 *vs* 8.97±5.96), and a significantly higher frequency of 16q-losses (*P*<0.001), 7p-gains (*P*<0.05) and combined 7p-gains/16q-losses (*P*<0.001). This cluster contained no tumours with strong (+++) overexpression of c-*erb*B2, p53 or expression of Ck 5/6.

## DISCUSSION

In recent years, cytogenetic progression models for various epithelial neoplasms have been postulated (Jiang *et al*, 2000; Boecker *et al*, 2001). However, straightforward statistical interpretation of the complex aberration patterns by conventional analysis of CGH-ratio-profiles provided many limitations. Contemporary bioinformatics procedures can better handle these complex patterns, which may provide a better understanding of mechanisms of carcinogenesis.

In general, the nuclear grade does not seem to change much during tumour progression. Well-differentiated DCIS are associated with invasive carcinomas of low nuclear grade (Holland *et al*, 1994), while recurrences of breast cancer closely resemble the cytological pattern of their primary (Millis *et al*, 1998). Despite these general rules, some cases exist with an obvious intratumoral heterogeneity of the nuclear grade, pointing to the possibility of an occasional progression in nuclear grade (Cserni, 2002). Based on our present data on invasive breast cancer cases, we are only able to draw indirect conclusions.

**Figure 3:**
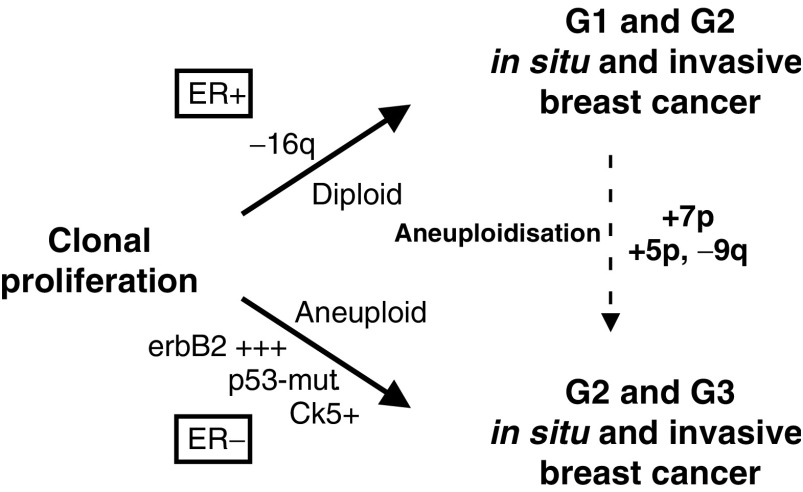
Extended hypothetical model of breast carcinogenesis with evidence for the existence of parallel and stepwise progression pathways. A limited subgroup of well-differentiated breast cancers, characterised by 16q-losses and ER-expression might progress towards poorly differentiated breast cancers, typically not belonging to the c-erbB2 overexpressing, Ck 5/6 expressing or p53 accumulating subgroups. This subgroup is characterised by the gain of chromosome 7p, and to a lesser extent by gains and losses of 5p and 9q, respectively. Whereas the step of progression is associated with aneuploidisation, the ER-expression remains stable. It will be a focus of further research work to define the frequency of transition between grades.

Since this series does not allow a prospective evaluation, the retrospective evaluation of ductal invasive grade 3 carcinomas seemed the most promising approach. This was carried out under the rationale that these tumours, as the extreme end of tumour de-differentiation, harbour 16q-losses in 20–30% of cases (Buerger *et al*, 1999a; Roylance *et al*, 1999). According to these results, a stepwise evolution via well-differentiated carcinomas could not be excluded theoretically for at least a subgroup of these tumours. From a cytogenetic point of view, these tumours seem to represent the extreme end of tumour de-differentiation with an accumulation of cytogenetic alterations that are rather rare in breast cancers of lower tumour grade. When subjecting the cytogenetic data to a biomathematical approach, alterations mainly seen in ductal invasive grade 3 cancers clearly formed different clusters (Figure 1). The importance of 13q-losses, 17q-gains and 20q-gains in breast cancers and their correlation with morphometric and other cytogenetic features have been discussed in detail before (Buerger *et al*, 2001). No data exist so far with respect to the meaning of 7p and 5p-gains or 9q-losses in breast cancer, but they are definitely part of far advanced and complex cytogenetic alteration patterns. The immunohistochemical profile of these tumours gave hints that especially 7p-gains are the major hallmark of this alteration pattern. Ductal invasive grade 3 carcinomas with 7p-gains were mainly aneuploid, displayed 16q-losses in 71% of the cases and displayed a high average number of cytogenetic alterations. Even more important was the oestrogen receptor status. Contrary to expectations for these morphologically far-advanced tumours, 84% of these tumours were oestrogen receptor positive, 70% were progesterone receptor positive and none revealed a strong c-*erb*B2 overexpression or p53 accumulation (hallmarks of gene amplification or mutations, respectively). The whole cytogenetic and immunohistochemical pattern of tumours with 7p-gains, and to a lesser extent 5p-gains as well as 9q-losses (see also cluster in Figure 2), therefore puts them forward as putative examples of poorly differentiated tumours originating from well-differentiated tumours that have acquired a high degree of cytogenetic instability (Figure 3

). Even though the exact overall number of tumours following this pathway cannot be reliably delineated from this approach, this assumption is further supported by the cluster analysis of breast carcinomas based on their immunohistochemical expression pattern. Including a multitude of markers besides ER and PR, similar hints for the meaning of 7p-gains could be shown. 7p-gains were again predominantly present in a cluster characterised by a low average number of cytogenetic alterations and a significantly higher frequency of 16q-losses. It is known that distinct chromosomal alteration patterns seem to be associated with distinct protein expression patterns (Isola *et al*, 1999) and this also seems to hold true for 7p-gains and its associated alterations. The responsible genes involved in this cytogenetic instability associated with 7p-gains are largely unknown. The epidermal growth factor receptor (EGFR), located on 7p12-13, is an unlikely candidate gene (Briand *et al*, 1996), since the overexpression of EGFR in invasive breast cancer is associated with oestrogen receptor negativity (Harris *et al*, 1992). Gene expression analysis studies were able to show that multiple ER-positive breast cancer groups exist, at least one with a very unfavourable prognosis (Perou *et al*, 2000). Further studies have to show if this specific cytogenetic subgroup with 7p-gains and a high degree of genetic instability represents the cytogenetic homologue for these ER-positive tumours defined by gene expression analysis. Especially, the finding of a high average number of cytogenetic alterations, shown by itself to be a bad prognostic marker (Isola *et al*, 1995), and the association with 9q-losses (Gray, 2001) make this likely.

In summary, our results on the one hand show that existing explanation models of breast carcinogenesis are at least partially verified by independent methods, but can also be extended by additional biomathematical procedures. With the introduction of other high-throughput methods such as tissue-arrays (Kononen *et al*, 1998; Korsching *et al*, 2002) for the determination of distinct protein expression patterns, or gene expression-profiling (Perou *et al*, 2000), it will be possible to gain a more complete picture of a single tumour, allowing a more detailed analysis of mechanisms in the pathogenesis of breast cancer.
